# Chronic Nonbacterial Osteomyelitis of the Sternocostoclavicular Region in Adults: A Single‐Center Dutch Cohort Study

**DOI:** 10.1002/jbm4.10490

**Published:** 2021-04-10

**Authors:** Ashna IE Ramautar, Natasha M Appelman‐Dijkstra, Shannon Lakerveld, Marielle A Schroijen, Marieke Snel, Elizabeth M Winter, Neveen AT Hamdy

**Affiliations:** ^1^ Centre for Bone Quality, Department of Medicine, Division of Endocrinology Leiden University Medical Center Leiden The Netherlands

**Keywords:** AUTOINFLAMMATION, AXIAL SKELETON, CHRONIC NONBACTERIAL OSTEITIS, HYPEROSTOSIS, PALMOPLANTAR PUSTULOSIS, SCLEROSIS

## Abstract

Sternocostoclavicular hyperostosis (SCCH) is a rare autoinflammatory bone disorder caused by chronic nonbacterial osteomyelitis (CNO), which is associated with sclerosis and hyperostosis primarily affecting the sternum, the medial end of the clavicles, and the first ribs. Other areas of the axial skeleton may also be affected. The more severe synovitis–acne–pustulosis–hyperostosis–osteitis (SAPHO) syndrome is additionally associated with dermatoses and joint manifestations. This Dutch retrospective cross‐sectional single‐center cohort study characterizes the spectrum of clinical features in adult CNO/SCCH patients at the time of diagnosis. The only inclusion criteria was the availability of complete sets of clinical and imaging data systematically collected over three decades using in‐house protocols. Data from 213 predominantly female patients (88%) with a median age of 36 years at presentation were studied. The mean diagnostic delay was 5 ± 5 years. The main symptoms were chronic pain (92%), bony swelling (61%), and restricted shoulder girdle function (46%); 32% had palmoplantar pustulosis and 22% had autoimmune disease. The majority (73%) had isolated SCCH; 59 (27%) had additional localizations in vertebrae (19%), the mandible (9%), or both (2%); 4 had SAPHO. The prevalence of current or past smoking was high (58%), particularly for patients with palmoplantar pustulosis (76%). There was a significant relationship between delay in diagnosis and both the extent of affected skeletal sites (*p* = 0.036) and erythrocyte sedimentation rate levels (*p* = 0.023). Adult‐onset CNO is characterized by distinctive clinical and radiological features, but diverse aspects of its spectrum are currently not fully captured by a comprehensive classification. Delayed diagnosis is still common and potentially associated with irreversible structural changes and debilitating chronic symptoms, increasing the burden of illness and negatively impacting on quality of life. It is hoped that findings from this study will dispel confusion about nomenclature and classification of adult‐onset CNO and increase awareness of its distinctive clinical and radiological features, and thus facilitate early diagnosis and referral for treatment, which should positively impact prognosis by preventing disease progression, although this remains to be established. © 2021 The Authors. *JBMR Plus* published by Wiley Periodicals LLC on behalf of American Society for Bone and Mineral Research.

## Introduction

Chronic nonbacterial osteomyelitis (CNO) is a rare autoinflammatory bone disorder, which leads to sclerosis and hyperostosis of affected sites of the axial skeleton in adults, and predominantly of the appendicular skeleton in children and adolescents.^(^ ^1^, ^2^, ^3^, ^4^ ^)^

The predilection of the lesions for skeletal sites at the sternum, the medial ends of clavicles, and the upper ribs has led to the coining of the descriptive term sternocostoclavicular hyperostosis (SCCH; Orphanet no. 178311). The clinical expression of CNO in children and adolescents is chronic recurrent multifocal osteomyelitis (CRMO; Orphanet no. 324964), with lesions predominantly localized in long bones, although any bone may be affected except for the neurocranium. Palmoplantar pustulosis (PPP) is the pathognomonic chronic sterile inflammatory dermatosis solely localized in the skin of the palms and the soles of feet, reported to occur before, during, or after the development of skeletal manifestations of CNO. Varying between series, this characteristic dermatosis is observed to occur in up to 60% of patients with CNO. ^(^ ^5^, ^6^, ^7^, ^8^, ^9^ ^)^

The more severe form of CNO in adults, coined the SAPHO syndrome (Orphanet no. 793) by Chamot in 1987, is characterized by the association of the cornerstone feature of osteitis with extraskeletal manifestations, mainly in the form of the characteristic dermatosis, PPP, and severe acne, and synovitis mainly affecting large joints.

Although CRMO cases were included in the original description of SAPHO syndrome, there is still no consensus about whether CNO/CRMO is a childhood form of SAPHO or whether it represents a separate entity based on its effects on different parts of the skeleton in children. ^(^ ^10^, ^11^ ^)^

The precise pathophysiology of CNO/SCCH remains elusive, although an imbalance in cytokine expression and secretion by immune cells, a high prevalence of autoimmune disease among patients and first‐degree relatives, and the identification of family clusters of SCCH suggest an autoimmune and genetic background for the disorder.^(^ ^1^, ^12^ ^)^ Opinions are still divided on the possible contribution of an initial infectious phase, possibly by a nonpathogenic organism such as *Propionibacterium acnes*,^(^ ^13^, ^14^, ^15^, ^16^, ^17^, ^18^, ^19^ ^)^ or altered gut microbiome, which may represent the trigger for the immune and inflammatory process.^(^ ^20^, ^21^ ^)^

Since its first description in the Japanese literature in the late 1960s,^(22)^ several names and different classification criteria have been used to describe the spectrum of the clinical manifestations of CNO, with the variable overlapping nomenclature resulting in confusion as to definitive diagnostic criteria, natural history, treatment options, and validated outcome parameters for treated disease. ^(^ ^7^, ^10^, ^11^, ^23^, ^24^, ^25^, ^26^, ^27^, ^28^ ^)^ This leads to the disease often going unrecognized for years, with delay in diagnosis resulting in chronic potentially debilitating symptoms, which we have previously shown to have significant impact on quality of life. ^(^ ^5^, ^29^ ^)^

The primary aim of this study was to highlight the spectrum of clinical features of adult‐onset CNO/SCCH by analyzing data collected over three decades from our large single‐center cohort of more than 200 adult patients with an established diagnosis of CNO/SCCH. The ultimate objective of the study was to dispel the current confusion in the nomenclature and classification of this disorder to facilitate early diagnosis and timely referral for treatment, which is particularly relevant for CNO/SCCH because delays in diagnosis have been clearly shown to increase the burden of illness.

## Materials and Methods

**Fig 1:**
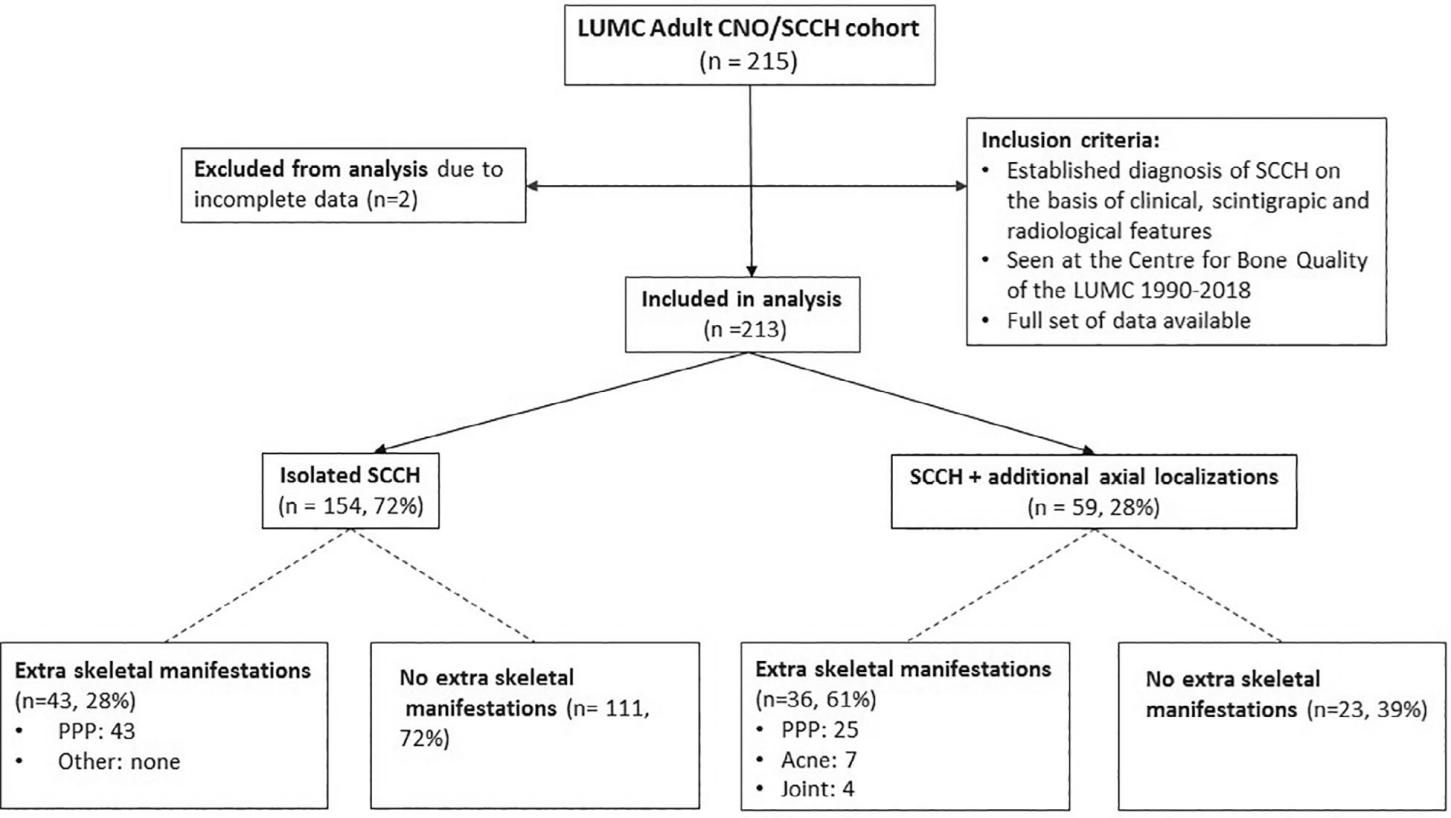
Study flow chart. CNO, chronic nonbacterial osteomyelitis; LUMC, Leiden University Medical Center; PPP, palmoplantar pustulosis; SCCH, sternocostoclavicular hyperostosis.

In this retrospective cross‐sectional cohort study, 213 adult patients with a diagnosis of CNO/SCCH based on characteristic clinical, scintigraphic, and radiological features were included in the study. All patients had their diagnosis established or confirmed between 1990 and 2018 at the Centre for Bone Quality of the Leiden University Medical Center, a Dutch national expertise center for bone and mineral disorders including rare bone disorders, and a full health care provider member of the European Reference Network for Rare Bone Diseases, ERN‐BOND. The availability of a complete set of demographic, clinical, and imaging data was a prerequisite for including data from individual patients in the study (Fig. 1).

**Fig 2:**
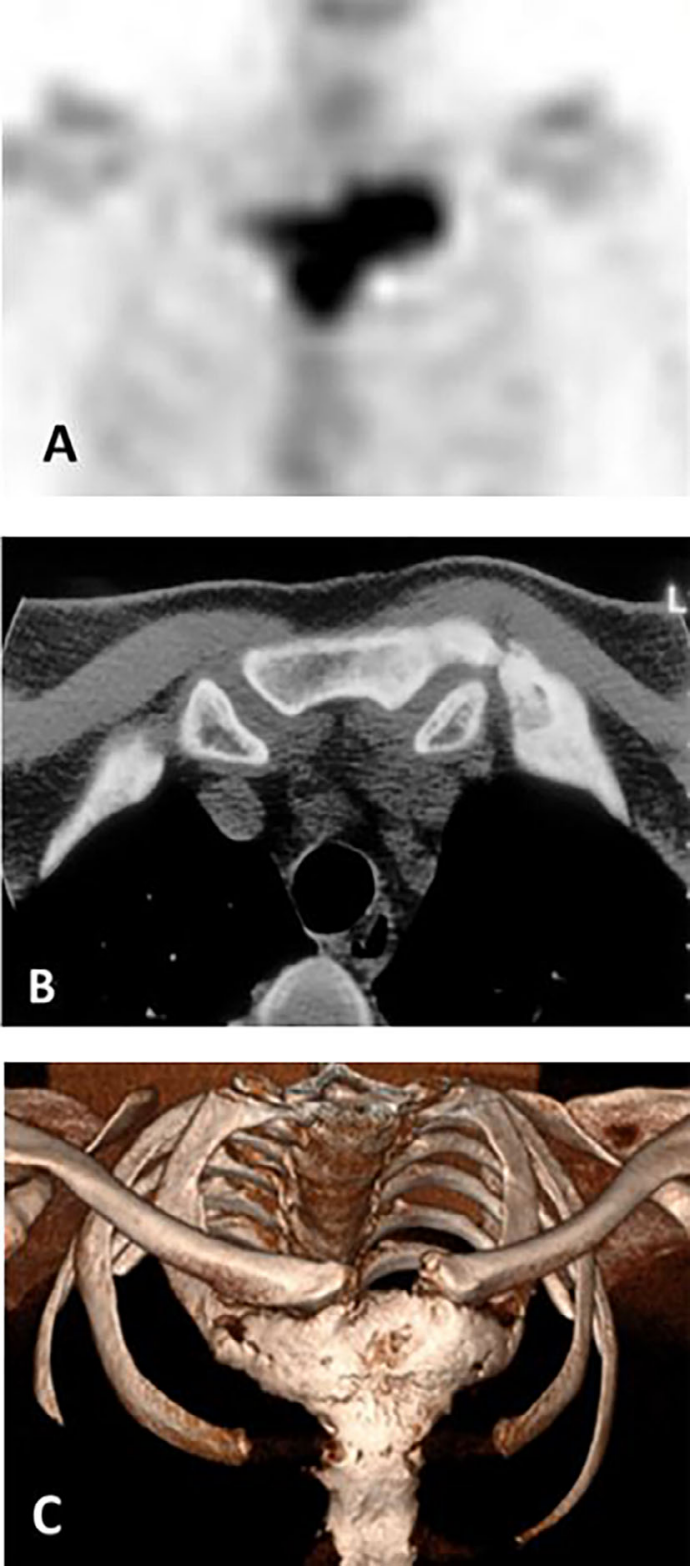
Distinctive imaging features of chronic nonbacterial osteomyelitis (CNO) of the sternocostoclavicular region in an adult patient with CNO/sternocostoclavicular hyperostosis (SCCH). (*A*) ^99m^Tc skeletal scintigraphy showing increased radioactive isotope uptake of the medial end of the clavicle, manubrium sternum, and first rib. (*B*) Distinctive radiological features of sclerosis and hyperostosis of affected bones on transverse CT imaging of the region in the same patient. (*C*) Three‐dimensional image reconstruction of a CT scan of the sternocostoclavicular region in a different patient with CNO/SCCH showing hyperostosis of the manubrium, medial end of the first rib on the left, and of the whole first rib and medial end of the clavicle on the right, forming a large bony costomanubrial bridge and ankylosis of the manubriosternal joint.

Data analyzed in this study had been systematically collected over the past three decades, using in‐house developed protocols, and were retrieved from medical records. The data included demographic information: sex, age at first symptom(s), and age at diagnosis; clinical information: symptoms and signs at presentation and at diagnosis, a family history of CNO, and presence of PPP and of autoimmune diseases; laboratory information at diagnosis: serum levels of inflammatory markers C‐reactive protein (CRP) and erythrocyte sedimentation rate (ESR), and bone turnover markers serum alkaline phosphatase (ALP) and type 1 procollagen N‐terminal (P1NP); and imaging information: skeletal distribution of lesions as evidenced by increased radioactive isotope uptake on 99 m‐technetium (^99m^Tc) bone scintigraphy, the presence of distinctive radiological features of sclerosis and/or hyperostosis on CT scans of the SCC region, and when required, plain radiography of the spine and sacroiliac region and/or mandible (Fig. 2). When available, data on shoulder function were also retrieved, as were data on the consequences of disease burden on the patient's ability to remain in the workforce.

The collection and analysis of data used in this study were approved by the Medical Ethics Committee of the Leiden University Medical Center.

### Statistical analysis

Statistical analysis was performed using SPSS for Windows, Version 23.0 (SPSS, Inc) Results are presented as mean (±SD) or as median (range) as applicable, and categorical variables were summarized using frequency counts and percentages. Comparison between data was made using unpaired *t* test or Mann‐Whitney *U* test for numerical data, and the χ^2^ test for categorical data. Level of significance was set at *p* < 0.05. Pearson's and Spearman's correlations were used for the assessment of correlations between clinical parameters and serum levels of inflammatory and bone markers for normally and nonnormally distributed variables, respectively.

## Results

### Whole cohort

**Fig 3:**
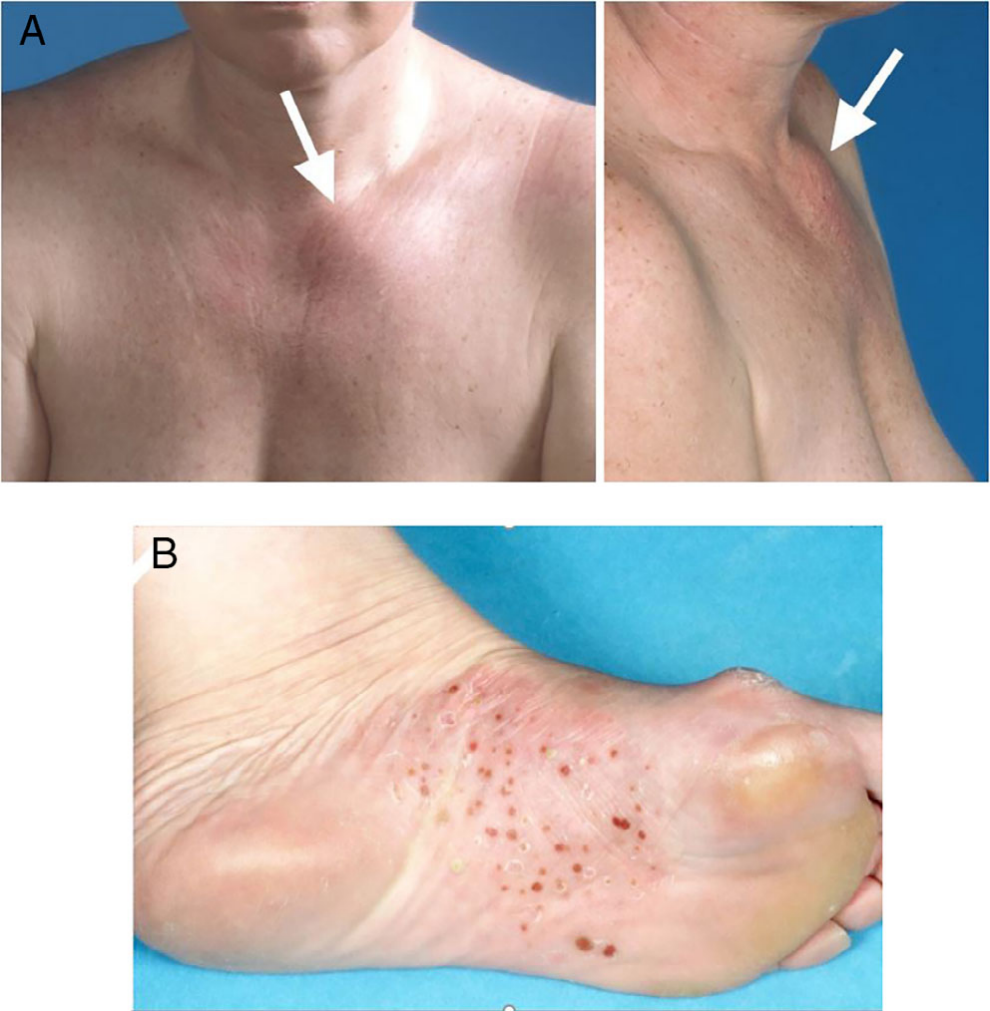
Clinical features of chronic nonbacterial osteomyelitis/sternocostoclavicular hyperostosis (CNO/SCCH). (*A*) Front and lateral photographs of a 36‐year‐old female patient with isolated SCCH and palmoplantar pustulosis, showing local inflammatory changes of the left sternoclavicular region of the anterior chest wall in the form of redness and swelling. (*B*) Typical skin lesions of the sole, characteristic of palmoplantar pustulosis.

The cohort consisted of 213 predominantly female patients (*n* = 188, 88%; Table 1). Median age at presentation with first symptom(s) was 36 years (range, 14–72 years). Main presenting symptom was pain in 205 patients (96%), predominantly localized in the SCC region in 181 patients (85%), followed by local inflammatory changes in 88 of patients (41%), mainly in the form of warmth, redness, and soft tissue swelling in 77 (36%). Fifty‐four patients (25%) had a visible bony swelling in the anterior chest wall (Fig. 3*A*) or mandible. At diagnosis, median age was 44 years (range, 17–73 years), with a mean delay in diagnosis of 5.2 ± 5.5 years. In the interval between first presentation and diagnosis, an additional 76 patients (36%) had developed local bone enlargement. Ninety‐seven patients (46%) had restriction of shoulder girdle function on the same side as the bony lesion. PPP was present in 68 (32%) of the cohort's 213 patients (Fig. 3*B*). Only four patients (2%) were asymptomatic—the diagnosis of CNO/SCCH having been established by evidence for increased radioactive uptake in one or more affected sites on skeletal scintigraphy and the presence of sclerosis with or without hyperostosis on CT scan of the SCC region.

**Table 1:** Demographic and Clinical Characteristics of Adult Patients With CNO/SCCH

Baseline characteristics	Whole cohort n = 213	Isolated SCCH n = 154	SCCH with other axial localizations n = 59	*p* Value	SCCH with extraskeletal manifestations n = 74	SCCH without extraskeletal manifestations n = 139	*p* Value
Age at first symptoms, y^a^	36 (14–72)	35 (16–70)	41(14–72)	**0.048** *	37 (15–72)	35 (14–70)	0.185
Age at diagnosis, y^a^	44 (17–73)	41 (17–73)	51 (18–72)	**0.014** *	46 (22–72)	43 (17–73)	**0.034** *
Delay in diagnosis^b^	5.2 (5.5)	5.0 (5.4)	5.5 (5.6)	0.599	6.0 (6.8)	4.7 (4.6)	0.120
Sex female/male, %	88/12	92/8	80/20	**0.016** *	82.4/17.6	91.4/8.6	0.054
Initial symptom^c.d^							
Pain	205 (96.2)	149 (96.1)	56 (98.2)	0.444	70 (95.0)	135 (97.1)	0.596
Impaired shoulder function	72 (33.6)	58 (37.2)	14 (24.6)	0.074	20 (27.0)	52 (37.4)	0.064
Acute inflammatory changes	88 (41.3)	61 (39.1)	27 (47.4)	0.250	26 (35.1)	62 (44.6)	0.158
Bone swelling	54 (25.3)	40 (25.6)	14 (24.6)	0.726	16 (21.6)	38 (27.3)	0.396
Fever	0 (0.0)	0 (0.0)	0 (0.0)	‐	0 (0.0)	0 (0.0)	‐
None	4 (1.9)	4 (2.6)	0 (0.0)	‐	0 (0.0)	4 (2.9)	‐
Physical examination at diagnosis^c^							
Pain	208 (98.0)	152 (97.4)	56 (98.2)	0.932	73 (98.6)	135 (97.1)	0.954
Impaired shoulder function	97 (45.5)	77 (49.4)	20 (35.1)	0.070	29 (39.2)	68 (48.9)	0.082
Acute inflammatory changes	70 (32.9)	54 (34.6)	16 (28.1)	0.398	17 (23.0)	53 (38.1)	0.255
Bone swelling	130 (61.0)	91 (58.3)	39 (68.4)	0.175	44 (59.5)	86 (61.9)	0.995
Fever	0 (0.0)	0 (0.0)	0 (0.0)	‐	0 (0.0)	0 (0.0)	‐
Presence PPP^c^	68 (31.9)	43 (28.0)	25 (42.4)	**0.043** *	68 (92.0)	0 (0.0)	‐
Smokers (status in n = 66)	50/66 (75.8)	31/42 (74.0)	19/24 (76.0)	0.625	50 (75.8)	0 (0.0)	‐
Smoking before/at diagnosis (status in n = 201)^c^	114/201 (56.7)	77/145 (53.0)	37/56 (66.1)	0.096	52/71 (73.2)	62/130 (47.7)	**0.001** *
PPP	50 (44.0)	31 (40.3)	19 (51.4)	0.264	50 (96.2)	0 (0.0)	‐
Acne (severe)^c^	7 (3.3)	*‐*	7 (11.9)	‐	7 (9.5)	0 (0.0)	‐
Joint involvement^c^	4 (1.9)	‐	4 (6.8)	‐	4 (5.4)	0 (0.0)	‐
Prevalence familial SCCHc	22 (10.3)	17 (12.0)	5 (10.2)	0.738	9 (12.2)	13 (9.4)	0.721
No. of families	7	5	2	‐	2	5	‐

*Note*. Data expressed as ^a^median (range), ^b^mean (SD), ^c^no. of patients (%), and ^d^more than one symptom possible.

Abbreviations: CNO, chronic nonbacterial osteomyelitis; PPP, palmoplantar pustulosis; SCCH, sternocostoclavicular hyperostosis.

* Significance at *p* < 0.05.

Bold text highlights statistically significant differences in demographic or clinical characteristics between patients with “isolated SCCH” compared to patients with “SCCH with other axial localisations” or to patients with “SCCH with extraskeletal manifestations”.

Analysis of prediagnosis detailed history data suggests that the inflammatory process is characterized in its early stages by recurrent episodes of exacerbation, as clinically expressed by local inflammatory changes in the form of warmth, redness, and soft tissue swelling at affected skeletal sites (Table 1). These episodes of exacerbation are initially of short duration, resolving spontaneously within 1 to 2 weeks, followed by longer periods of remission. The natural course of the disorder suggests that with time, periods of exacerbation become longer and periods of remission shorter, until manifestations become chronic, associated with intermittent worsening caused by the recurrence of flares of inflammation.

Data on smoking status were available at diagnosis in 201 of the 213 patients, of whom 114 (58%) were current or past smokers, some for a high number of pack‐years. One‐hundred fourteen of the 213 patients (54%) were employed at the time of diagnosis, 31 of whom (27%) had chronic complaints resulting in reduced capacity to work, with 5 having to stop working because of debilitating chronic symptoms. Disease clustering was observed in seven families of known patients, who had two or more affected family members (*n* = 22; Table 1). One or more autoimmune disorders were present in 46 patients (22%), the most prevalent being psoriasis vulgaris in 10% of patients. The presence of at least one autoimmune disease was also documented in first‐degree relatives of 94 patients (44%): rheumatoid arthritis being the most prevalent in family relatives of 50 patients (24%; Table 3).

Imaging investigations used to establish a cause for the skeletal manifestations of the disorder before referral included CT scans (68%), ^99m^technetium bone scans (47%), MRI (16%), plain radiographs (8%), and PET scans (2%). Three or more diagnostic modalities had been performed in more than 30% of patients.

Despite not having an established diagnosis, the majority of patients had been empirically treated with nonsteroidal anti‐inflammatory drugs (62%), and one in five (21%) had received various oral or intravenously administered bisphosphonates using a variety of schedules prior to referral to our center. A minority had also been treated with biologicals such as anti‐TNFα (3%) or disease‐modifying antirheumatic drugs such as methotrexate or sulphasalazine (11%) as first‐line of treatment. Other treatment modalities included intra‐articular shoulder injections in 38 patients (18%), resection of the sternoclavicular joint in 5 patients (2%), decortication of the mandible for severe symptomatic hyperostotic lesions in 5 patients (2%), and 2 patients (1%) had been treated with antibiotics. Prior to referral, 31% of patients received no treatment despite having been symptomatic, sometimes for a number of years.

Compared with a mean delay in diagnosis of 5.6 ± 5.9 years when our then only 52 patient cohort was evaluated more than a decade ago, mean delay in diagnosis for the whole cohort was 5.2 ± 5.5 years, breaking down into >5 years in 33%, 3 to 5 years in 26%, and <3 years in 41% of patients.

In the whole cohort, the skeletal site most commonly affected was the clavicle in 76% (n = 162) of cases, followed by the first and second ribs in 60% (n = 129), and the sternum in 50%. Skeletal lesions were isolated to the SCC region in 154 patients (73%), and 59 patients (27%) had additional affected areas of the axial skeleton such as spine, pelvis, or mandible.

**Fig 4:**
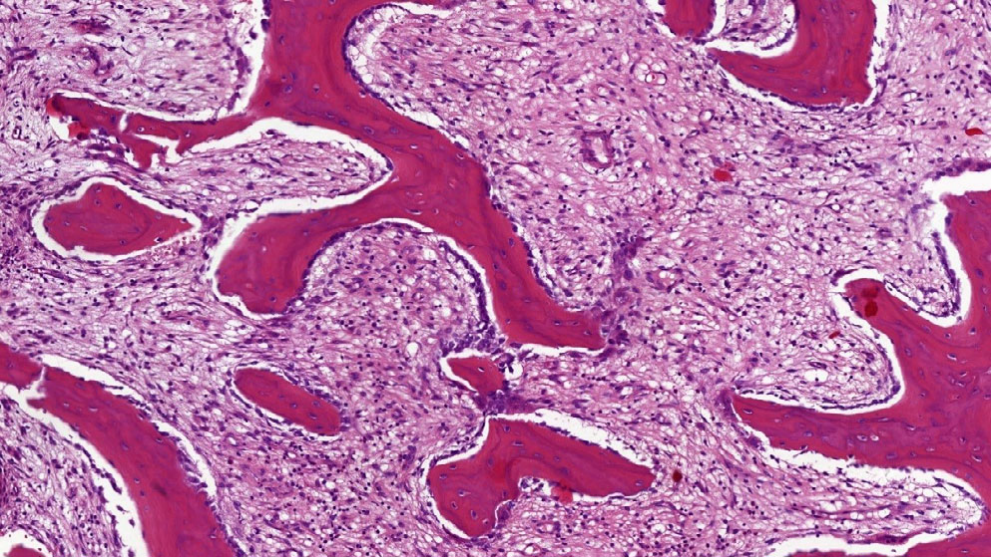
Hematoxylin and eosin‐stained bone biopsy of the medial end of the clavicle of a 45‐year‐old female patient showing a nonspecific chronic inflammatory cellular response with infiltration of the bone marrow with mixed inflammatory cells, predominantly monocytes and lymphocytes, increased osteoblastic activity, and bone marrow fibrosis.

Twenty‐eight patients (13%) had a bone biopsy performed elsewhere before referral, mostly to rule out a malignant bone tumor. In all cases, bone histology was reviewed in our center and showed features of a nonspecific inflammatory cellular response with cellular infiltration of the bone marrow with predominantly monocytes and granulocytes in the early stages and monocytes and lymphocytes, increased osteoblastic activity, and areas of osteolysis and fibrosis of the marrow in later stages (Fig. 4).

In the whole cohort, mean value of standard hematological and biochemical investigations (full blood count, renal and liver function, electrolytes, and 25(OH)D) were within the normal laboratory reference range at diagnosis. Serum concentrations of inflammatory markers and bone turnover markers were also within the normal reference range in 91% and 82% of patients, respectively, and were otherwise only mildly elevated at diagnosis. Mean serum levels of CRP (normal <5.0 mg/L) and ESR (normal <20 mm/h) were respectively slightly above normal and normal, mean 7.3 mg/L (SD, 8.5 mg/L) and 18.3 mm/h (SD, 18.6 mm/h). There was a significant correlation between extent of skeletal lesions as documented by 99mTc skeletal scintigraphy and ESR and CRP levels (*p* < 0.001 and *p* = 0.002, respectively), and between disease duration and ESR levels (*p* = 0.023; Table 5). Mean concentration of serum markers of bone turnover was not elevated at diagnosis: ALP = 80.7 ± 30.2 U/L (normal <98 U/L) and P1NP = 45.4 ± 19.6 ng/ml (normal <59 ng/ml). Only a limited number of patients (n = 56 of 213, 26%), were tested for the HLA‐B27 antigen, with only seven testing positive, two of whom had radiological evidence for a spondyloarthropathy.

### Clinical characteristics of CNO subtypes

#### Isolated sternocostoclavicular hyperostosis

One hundred fifty‐four of the 213 cohort patients (73%) had isolated SCCH. The majority were women (*n* = 141, 92%), with a sex ratio of 12:1 (Table 1). Median age at first symptom(s) was 35 years (range, 16–70). Pain in the affected axial sites of the anterior chest wall was the most common initial symptom in 130 patients (84%), followed by local acute inflammatory changes in 61 patients (39%), mainly in the form of local soft tissue swelling (n = 54, 35%), and a bony swelling in 40 patients (26%), predominantly unilaterally affecting the medial end of a clavicle.

Median age at diagnosis was 41 years (range, 17–73 years), with a mean delay in diagnosis of 5.0 ± 5.4 years from appearance of first symptom. In the interval between initial symptoms and diagnosis, an additional 73 patients (47%) developed a bony swelling at the SCC region, bringing the total number of patients with isolated SCCH having a bony swelling at diagnosis to 113 patients (72%). At the time of diagnosis, a limitation of shoulder girdle function was observed unilaterally on the same side as the lesion, or bilaterally in 77 patients (49%). Four patients (2%) were asymptomatic, with the diagnosis established in the course of family screening for SCCH.

Smoking status was available in 145 of the 154 patients, 77 of whom (53%) had been smoking prior to or at diagnosis. Disease clustering was observed in families of five known patients (Table 1).

Diagnostic imaging showed that the most commonly affected skeletal site was the medial end of the clavicle (mainly unilateral) in 79% (n = 123), followed by the first and second ribs in 58% of patients (n = 91). The sternum was also affected in 52% of patients. Solitary lesions were present in 26 patients (17%), including the clavicle in 17 patients, a rib in 7 patients, and the sternum in 2 patients (1%). Sixty‐seven percent of patients (*n* = 104) had more than one affected site in the SCC region (Table 2).

**Table 2:** Distribution of Skeletal Lesions of CNO/SCCH Based on ^99m^Tc Scintigraphy and CT Scans

Affected sites	Whole cohort *n = 213(%)*	Isolated SCCH *n = 154(%)*	SCCH with other axial localizations *n = 59(%)*
Anterior chest wall	213 (100.0)	154 (100.0)	59 (100.0)
Clavicle (medial end)	162 (76.0)	123 (78.8)	39 (68.4)
Bilateral	74 (34.6)	55 (35.3)	19 (33.3)
Unilateral	88 (41.1)	68 (43.6)	20 (35.1)
Ribs	129 (60.3)	91 (58.3)	38 (66.7)
Bilateral	81 (37.9)	56 (35.9)	25 (43.9)
Unilateral	48 (22.4)	35 (22.4)	13 (22.8)
Sternum	107 (50.0)	81 (51.9)	26 (45.6)
Spine	40 (18.8)	‐	40 (70.2)
Cervical spine	6 (2.8)	‐	6 (10.5)
Thoracic spine	6 (2.8)	‐	6 (10.5)
Lumbar spine	10 (4.7)	‐	10 (17.5)
Multiple levels of the spine	13 (6.1)	‐	13 (22.8)
Sacrum/Iliac bone	5 (2.3)	‐	5 (8.8)
Mandible	20 (9.4)	‐	20 (35.1)
Bilateral	4 (1.9)	‐	4 (7.0)
Unilateral	16 (7.5)	‐	16 (28.1)
Mandible and spine	4 (1.9)	‐	4 (7.0)
>1 affected site of the axial skeleton	161 (74.2)	104 (66.7)	59 (100.0)
≥3 affected site of the axial skeleton	76 (35.7)	34 (21.8)	42 (73.7)

Abbreviations: CNO, chronic nonbacterial osteomyelitis; SCCH, sternocostoclavicular hyperostosis.

PPP was present in 43 patients (28%). A diagnosis of one or more autoimmune diseases was established in 36 patients (23%), the most prevalent being psoriasis vulgaris in 36% (n = 13). The prevalence of at least one autoimmune disease was documented in first‐degree family relatives of 67 patients (44%), with rheumatoid arthritis being the most prevalent in family relatives of 30 patients (19%; Table 3).

**Table 3:** Prevalence of Autoimmune Disease in Patients and First‐Degree Relatives

Autoimmune disease	Patients n = 213 (%)	No. of patients with affected first‐degree relatives (%)
Any autoimmune disease^a^	46 (22.0)	94 (44.1)
Psoriasis vulgaris	21 (9.7)	19 (8.9)
Rheumatoid arthritis	2 (0.9)	50 (23.5)
Ankylosing spondylitis	9 (4.2)	6 (2.8)
Polymyalgia rheumatica	2 (0.9)	2 (0.9)

^a^ Includes one or more autoimmune disease.

Mean serum levels of inflammatory and bone markers were normal in 96% and 82% of patients, respectively. Mean serum levels for CRP and ESR were within the normal range, at 6.4 ± 7.0 mg/L and 16.1 ± 18.3 mm/h, respectively. In this subgroup, there was no relationship between the level of inflammatory markers and disease duration or number or extent of skeletal lesions. Mean serum levels of bone turnover markers were not elevated: 79.1 ± 27.6 U/L (normal <98 U/L) for ALP and 43.0 ± 20.2 ng/ml (normal <59 ng/ml) for serum P1NP.

Only 5 of the 44 patients who were tested for HLA‐B27 antigen in this subgroup were found to be positive, without showing radiographic evidence for spondyloarthritic spinal changes.

#### SCCH with additional axial localizations

Fifty‐nine of the cohort's 213 patients (27%) had additional axial localizations (no separate Orphanet no.). There were 45 women (79%) and 12 men (21%) with a sex ratio of 3.8:1 (Table 1). All patients in this subgroup were symptomatic, and median age at first symptom(s) was 41 years (range, 14–72 years). Median age at diagnosis was 51 years (range, 18–72 years), with a mean delay in diagnosis of 5.6 ± 5.7 years. Forty patients (70%) had one or more vertebral lesions, although only 19 had back pain of variable severity. Thirteen patients (23%) had lesions at multiple levels of the spine, the cervical and lumbar spine being the most commonly affected in 19 and 23 patients, respectively.

Twenty patients (35%) had a mandibular localization, which was predominantly unilateral in 80%. All patients with a mandibular lesion had a painful swelling at the affected site of the jaw at time of diagnosis. Seven had hypertonia of the jaw musculature; five had functional disturbance in the form of trismus. Four patients had both spine and mandibular localizations (Table 2).

Smoking status was available in 56 of the 59 patients, 37 of whom (66%) had been smoking prior to or at the time of diagnosis. Disease clustering was observed in two families of known patients (Table 1). PPP was present in 25 patients (43%), and one or more autoimmune disease had been diagnosed in 11 patients (19%): the most prevalent being psoriasis vulgaris in 7% (n = 4). The presence of at least one autoimmune disease was also documented in first‐degree relatives of 27 patients (46%), with rheumatoid arthritis being the most prevalent in relatives of 16 of the 59 patients with this more extensive disease (29%; Table 3).

**Fig 5:**
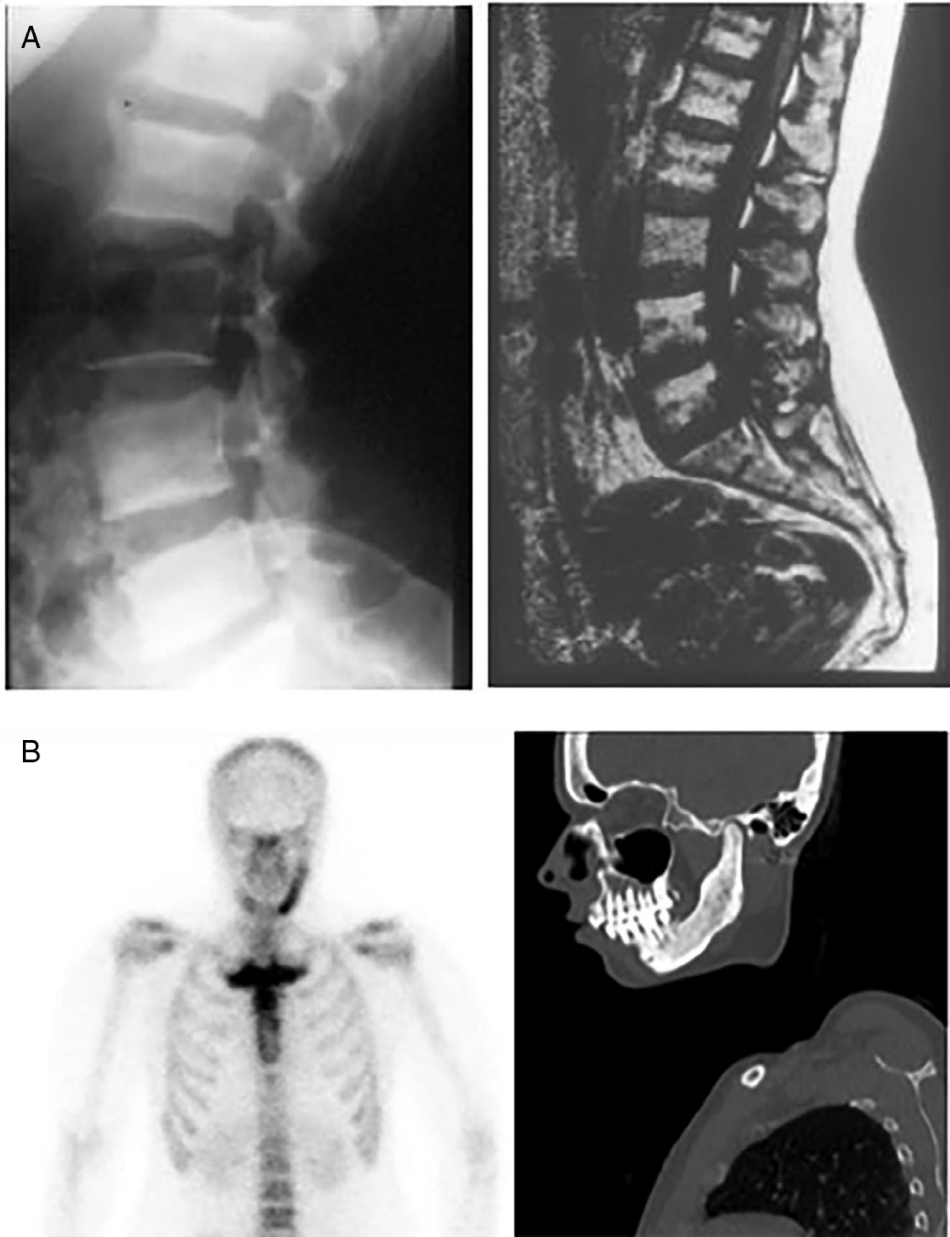
(*A*) Other axial localizations in chronic nonbacterial osteomyelitis/sternocostoclavicular hyperostosis (CNO/SCCH); multiple level vertebral involvement in a patient with CNO/SCCH showing irregular sclerosis of multiple vertebrae especially at the end plates and starting ossification of anterior ligaments, with only the third lumbar vertebra being spared on plain lateral radiographs of the spine on the left, as also confirmed on MRI on the right. (*B*) Mandibular localization of chronic nonbacterial osteomyelitis.

Mean serum levels of CRP and ESR were within the normal reference range at 9.8 ± 11.4 mg/L and 24.0 ± 18.5 mm/h, respectively, with only 17% of patients having CRP or ESR levels slightly above the normal reference range. There was no relationship between serum level of inflammatory markers and disease extent or severity as judged by the number of affected sites on bone scintigraphy. Mean level of bone turnover markers, ALP, and serum P1NP were within the normal laboratory reference range at 84.8 ± 36.0 U/L and 46.0 ± 17.8 ng/ml, respectively (Table 4). We observed a significant correlation between number of skeletal lesions and serum ALP activity (*p* = 0.019) in this subtype, reflecting a greater extent of skeletal involvement compared with patients with isolated SCCH. Sclerosis and/or hyperostosis of one or more vertebrae was observed in 40 of the 59 patients with spinal involvement (68%), with 13 patients (35%) having multiple affected vertebrae (Fig. 5, Table 2). Twenty patients with SCCH also had mandibular involvement (diffuse sclerosing osteomyelitis) and only four patients had the full features of SAPHO syndrome (Table 1).

**Table 4:** Serum Levels of Inflammatory and Bone Turnover Markers in Patients Grouped According to Type of CNO/SCCH

	Whole cohort *n* = 213	Isolated SCCH *n* = 154	SCCH with other axial lesions *n* = 59	*p* Value	SCCH with PPP *n* = 68	SCCH without PPP *n* = 145	*p* Value
Inflammatory markers^a^							
ESR (normal <20 mm/h)	18.3 (18.6)	16.1 (18.3)	24.0 (18.5)	**0.001** *	24.2 (22.8)	15.6 (15.7)	**0.001** *
CRP (normal <5.0 mg/L)	7.3 (8.5)	6.4 (7.0)	9.8 (11.4)	**0.044** *	6.5 (6.5)	7.7 (9.3)	0.821
Bone markers (α)							
ALP (normal <98 U/L)	80.7 (30.2)	79.1 (27.6)	84.8 (36.0)	0.367	81.8 (31.2)	80.1 (29.8)	0.387
P1NP (normal <59 ng/ml)	45.4 (19.6)	43.0 (20.2)	46.0 (17.8)	0.823	48.1 (19.8)	44.3 (19.5)	0.283
CTX (normal <0.573 ng/ml)	0.310 (0.144)	0.307 (0.156)	0.318 (0.121)	0.657	0.357 (0.071)	0.304 (0.15)	0.355

Abbreviations: ALP, alkaline phosphatase; CNO, chronic nonbacterial osteomyelitis; CRP, C‐reactive protein; CTX, carboxy‐terminal cross‐linking telopeptide of type 1 collagen; ESR, erythrocyte sedimentation rate; P1NP, type 1 procollagen N‐terminal; PPP, palmoplantar pustulosis; SCCH, sternocostoclavicular hyperostosis.

Bold text highlights statistically significant differences in serum levels of inflammatory markers between patients with “isolated SCCH” compared to patients with “SCCH with other axial lesions” or those with “SCCH with PPP”.

^a^ Expressed as mean (SD).

* Significance *p* < 0.05.

HLA‐B27 status was determined in 12 patients, 2 of whom had radiological changes for a spondyloarthropathy and tested positive, with only one of the two with symptoms of lower back pain.

#### Extraskeletal manifestations of SCCH and the SAPHO syndrome

Extraskeletal manifestations were present in 74 of the 213 (35%) cohort patients, of whom 68 (32%) had PPP, without specific chronological order with respect to skeletal manifestations, presenting before, during, or after onset of skeletal symptoms. Nine (4%) had severe acne, and four (1%) had additional joint involvement and the full features of the SAPHO syndrome. Patients in this subgroup were predominantly female (*n* = 61, 82%), with a sex ratio of 5:1 (Table 1). Median age at first symptom(s) was 37 years (range, 15–72 years). Median age at diagnosis was 46 years (range, 22–72 years), with a mean delay in diagnosis of 6.0 ± 6.8 years from first symptom. Patients in this group were significantly older at diagnosis and had more chronic symptoms than patients without extraskeletal manifestations (*p* = 0.034); 78% had more than one affected site of the axial skeleton, with 39% having three or more affected sites.

Seventy‐six percent of all patients with PPP were current or past smokers. Of the 114 smokers in the whole cohort, 50 patients (44%) had PPP compared with the low prevalence of PPP documented in the 16 patients with SCCH who never smoked (24%).

Among the 77 patients with isolated SCCH, who smoked before or at time of diagnosis, 31 (40%) had PPP compared with its presence in only 12 of the 43 patients with isolated SCCH who never smoked (26%). Among the 37 patients with SCCH and other axial localizations who were smoking before or at time of diagnosis, 19 (51%) had PPP. In contrast, among the 71 of the 74 patients with extraskeletal manifestations in whom smoking status was retrievable, 52 (73%) smoked before or at time of diagnosis, and 50 of the 52 smokers (96%) also had PPP. Nine of the 59 patients (15%) with SCCH and additional axial localizations had severe acne; four also had joint manifestations.

Thirteen patients (18%) with extraskeletal manifestations had one or more autoimmune disease, the most prevalent being psoriasis in 10% of patients (n = 7); 33 patients (45%) had one or more first‐degree relatives with an autoimmune disease, mainly rheumatoid arthritis (Table 3).

Mean levels of markers of bone turnover, ALP and serum P1NP were not elevated in patients with extraskeletal manifestations at 81.8 ± 31.2 U/L (normal <98 U/L) and 48.1 ± 19.8 ng/ml (normal <59 ng/ml), respectively. There was no significant difference in serum levels of bone turnover markers between patients with or without extraskeletal manifestations. However, there was a significant correlation between extraskeletal manifestations and smoking (*p* < 0.001) and ESR levels (*p* = 0.007; Table 5). Only four patients fulfilled all the SAPHO criteria of inflammatory synovitis, severe acne, pustulosis, hyperostosis, and osteitis.

**Table 5:** Correlations Between Serum Levels of Inflammatory and Bone Turnover Markers and Clinical Features (Whole Cohort)

	ESR		CRP		ALP		P1NP		CTX	
	R	*p* Value	R	*p* Value	R	*p* Value	R	*p* Value	R	*p* Value
Extent of affected skeletal sites	**0.285** ^*^	**0.000**	**0.276** ^*^	**0.002**	**0.168** ^**^	**0.019**	0.090	0.282	−0.016	0.942
Palmoplantar pustulosis	**0.191** ^*^	**0.007**	−0.041	0.660	0.019	0.792	0.094	0.257	0.250	0.238
Delay in diagnosis	**0.166** ^**^	**0.023**	0.134	0.152	0.026	0.726	−0.029	0.693	0.734	0.726

Abbreviations: ALP, alkaline phosphatase; CRP, C‐reactive protein; CTX, carboxy‐terminal cross‐linking telopeptide of type 1 collagen; ESR, erythrocyte sedimentation rate; P1NP, type 1 procollagen N‐terminal.

Bold text highlights statistically significant correlations between serum levels of inflammatory or bone turnover markers and extent of affected skeletal sites, presence of palmoplantar pustulosis, or delay in diagnosis.

^*^ Significance *p* < 0.01 (2‐tailed).

^**^ Significance *p* < 0.05 (2‐tailed).

### Comparison of clinical features between CNO subtypes

Patients with isolated SCCH were significantly younger at first symptom (*p* < 0.048) and at diagnosis (*p* < 0.014) than patients with more extensive axial lesions (Table 1), although there was no significant difference in delay in diagnosis between patients with isolated SCCH and those with additional axial localizations (mean 5 ± 5.4 and 5.5 ± 5.6), respectively. The prevalence of PPP was significantly higher in patients with one or more additional affected sites of the axial skeleton compared with patients with isolated SCCH (42% vs 28%, *p* = 0.043, respectively). Irrespective of subtype of SCCH, the presence of PPP appeared to be associated with a longer delay in diagnosis albeit nonsignificant (mean ± SD 6.0 ± 6.8 years and 4.7 ± 4.6 years, respectively; *p* = 0.12; Table 1). Significantly higher ESR but not CRP values were observed in patients with additional axial bone lesions (24.0 vs 16.1 mm/h, *p* = 0.001), Significantly higher ESR but not CRP values were also observed in patients with PPP (24.2 vs 15.6 mm/h, *p* = 0.001) compared with those with isolated SCCH or those with no PPP (Table 4).

## Discussion

Findings from this retrospective cross‐sectional cohort study characterize the spectrum of clinical features of CNO and its subtypes in a relatively large cohort of 213 adult patients, in whom data were systemically collected at diagnosis over three decades in a single Dutch center. To the best of our knowledge, our Leiden cohort represents one of the largest cohorts of adult patients with an established diagnosis of CNO so far reported. The literature on adult CNO/SCCH is relatively limited compared with the much more prolific pediatric CNO/CRMO literature, as summarized in a recent review publication by Hedrich and colleagues, as well as in a published report from the Eurofever International Registry, which included data on 486 patients: 355 children/adolescents with CNO/CRMO and 31 adults with CNO. ^(^ ^1^, ^3^ ^)^

Data from our relatively large cohort of adult patients with CNO/SCCH are largely in keeping with previously published data on adult CNO,^(^ ^2^, ^30^ ^)^ confirming the central diagnostic feature of the exclusive localization of skeletal lesions in the axial skeleton, predominantly in the SCC region, although less frequently also affecting vertebrae, the sacrum, and/or the mandible. Our data also confirm the previously reported difference in clinical features between adult patients with CNO and the more extensively studied patients with pediatric CNO, where the predominant localization of lesions is in long bones, although less frequently also affecting the SCC region, and other areas of the axial skeleton, including the mandible.^(^ ^1^, ^2^, ^3^, ^4^, ^7^, ^20^, ^26^ ^)^

In addition to the relatively large size of the cohort studied, one of the main strengths of our study is that in contrast to most published literature on CNO, which reports data mostly obtained in pediatric patients or in mixed populations consisting of mainly pediatric patients (CRMO) with a small number of adult patients, our cohort consists of strictly adult CNO patients—all having in common affected sites in the SCC region (SCCH). A further strength of our study is the availability of complete sets of demographic, clinical, scintigraphic, and radiographic data for the 213 adult patients represented in our cohort, which were collected over a span of three decades.

We are confident in the validity of our data, as although retrospective, which may be construed as a limitation of the study by the nature of limitations associated with retrospective studies, collection of the data was systematic, uniformly using standard in‐house developed protocols, including prediagnosis historical data. Collected data were accurately recorded in patients' medical files, and as mentioned above, only patients with complete data sets were included in the study.

**Fig 6:**
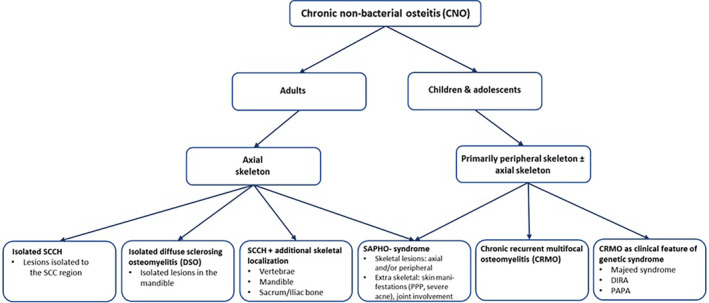
Proposed clinical classification of the spectrum of chronic nonbacterial osteomyelitis (CNO) in children and adults. SCCH, sternocostoclavicular hyperostosis; SCC, sternocostoclavicular region; SAPHO, synovitis, acne, pustulosis, hyperostosis, osteitis; DIRA, deficiency of interleukin‐1 receptor antagonist; PAPA, monogenic autoinflammatory syndrome characterized by sterile erosive arthritis, cystic acne, and pyoderma gangrenosum‐like lesions.

Our clinical and imaging findings at diagnosis, in a relatively large number of adult CNO patients, allowed us to identify and outline three distinct but also overlapping subtypes of adult CNO, as all having in common lesions in the SCC region (SCCH). These subtypes include isolated CNO/SCCH (identified by the separate Orphanet no. 178311); SCCH with additional skeletal localizations (no current Orphanet no.); and the more severe SAPHO syndrome (identified by the separate Orphanet no. 793). We propose to use our data highlighting the subtype characterization of the spectrum of CNO/SCCH to suggest a new classification of adult CNO based on the identified clinical subtypes (Fig. 6).

Another strength of our study is the availability of prediagnosis data, which were systematically recorded in patients' medical files at their first visit to our center. These data allowed us to calculate the timespan between the first symptom and establishment of diagnosis, confirming the continuing prevalence of delay in the diagnosis of CNO and its importance in determining the severity of disease at diagnosis. The timespan of the collection of the data, covering three decades, also led us to observe a decrease in the age of patients at diagnosis and a trend for shortening diagnostic delay between data reported more than a decade ago in the initial 52 patients of our cohort and data collected over the past decade.^(^ ^5^ ^)^ This latter observation suggested a potentially promising increased awareness for the disorder among treating physicians, leading to earlier referral to our expertise center for CNO for further evaluation and treatment. However, our data also suggested that delay in diagnosis is still prevalent, and that the disease is still unrecognized or misdiagnosed. This is likely based on the pattern of natural history of the disease, characterized by recurrent episodes of exacerbation and remission in its early stages, which often lead to significant delays before diagnosis is finally established, when chronic changes may no longer be reversible. Whereas some long‐term follow‐up data in adult patients with CNO/SCCH suggested that this disease may generally have a good prognosis,^(^ ^30^, ^31^, ^32^ ^)^ findings from our cohort suggested that the disease is nevertheless, not infrequently associated with a significant burden of illness caused by irreversible changes in the structure of affected bones, and consequent increased risk for secondary arthritis in adjacent joints, both leading to potentially debilitating chronic symptoms, impaired shoulder girdle function, and psychological and socioeconomic consequences. All these outcome features have significant impact on various aspects of quality of life, especially when diagnosis is delayed.^(^ ^5^ ^)^ In an earlier publication of data from the first 52 patients from our adult CNO cohort, we have shown that these poor outcome features were clearly linked with an extended interval between first clinical manifestation of SCCH and a confirmed diagnosis, leading to a delay in referral and in timely initiation of treatment.

Interestingly, the majority of patients in our adult CNO cohort had isolated SCCH. Both age at presentation and age at diagnosis were significantly higher when CNO also affected other axial localizations, possibly suggesting that the inflammatory process may be progressive, with further axial localizations being affected as patients grow older and remain untreated or inadequately treated, although this notion remains to be explored in long‐term placebo‐controlled follow‐up studies. Although there was no difference in age at first presentation between patients with and without extraskeletal manifestations, there was a significant difference in age at diagnosis between these two groups (*p* = 0.034). The presence of PPP was observed in all forms of CNO/SCCH, but this dermatosis was significantly more prevalent in patients with extensive axial localizations (*p* = 0.04). This suggested that the inflammatory process associated with PPP may contribute and/or exacerbate the severity of the inflammatory osseous manifestations of the disease. ESR levels were significantly correlated with the presence of PPP and with the extent of affected axial skeletal sites. Although mean levels of the bone formation markers ALP and P1NP were within the normal laboratory reference range in all subgroups, there was a significant correlation between number of affected skeletal sites and ALP activity, supporting the notion of the characteristic osteogenic response to inflammation observed in CNO. The consistently normal levels of bone turnover markers ALP and P1NP in the more focal isolated SCCH indicated that these markers should not be used for diagnosis, to assess disease activity or disease progression, or to monitor outcome of treatment, although these should be measured as part of the diagnostic process to exclude other bone pathology.

The primary pathology of CNO is a sterile (auto)inflammatory osteitis of affected bones of the SCC region and other areas of the axial skeleton or primarily one or more sites in long bones of the extremities in children, although axial sites may also be affected in this age group.^(^ ^1^ ^)^ Although the precise pathophysiological process of the autoinflammatory osteitis remains to be fully elucidated, recent evidence suggests an imbalance in the expression and secretion of proinflammatory cytokines such as IL‐6, IL‐20, and TNF‐α and antiinflammatory cytokines such as IL‐10 and IL‐19 by innate immune cells such as monocytes isolated from peripheral blood of patients with CNO/CRMO.^(^ ^1^, ^20^, ^33^ ^)^ The contribution of immunity to the pathophysiology of the disorder is further supported by the documented increase in the prevalence of autoimmune disease in patients with CNO/SCCH and their first‐degree relatives.^(^ ^12^ ^)^ A genetic background to the disorder is suggested by our documentation of previously unreported clustering of the disease in families of seven adult patients with CNO/SCCH. The possible contribution of an infectious nonpathogenic organism based on alterations in the skin microbiome such as *Propionibacterium acnes* or the gut microbiome as trigger for the altered immune response and for the aseptic inflammatory process remains controversial.^(^ ^13^, ^14^, ^15^, ^16^, ^18^, ^19^ ^)^ Cigarette smoking has been shown to play a role in inflammatory diseases such as Crohn disease, and to be a trigger and a source of exacerbation of other autoimmune diseases. The high number of current or past smokers among our CNO cohort is intriguing, but in keeping with the inflammatory nature of CNO, with the high prevalence of autoimmune disease in CNO patients, and with the established association between smoking and PPP.^(^ ^8^, ^34^, ^35^, ^36^ ^)^ It is noteworthy that although 44% of the 114 documented smokers in our cohort did not develop PPP, 76% of patients with PPP were smokers and a strong positive correlation was found between PPP and smoking (*p* < 0.001). Despite the analogy of the osteogenic response to inflammation in CNO, with that observed in ankylosing spondylitis, the limited number of patients of our cohort tested for HLA‐B27 precluded an adequate interpretation of a possible role for this antigen in the pathophysiology of CNO.

Although our study was not an epidemiological study, a number of epidemiological features could be gleaned from our retrospective data analysis. CNO/SCCH showed a clear female preponderance. First presentation was more common in young to midadult life, at a median age of 36 years compared with a median age of 42 years reported in our first series of 52 patients, suggesting that we may be capturing patients at an earlier stage of the disease. This premise is at least partly supported by the trend for a shortened period of diagnostic delay from an initial mean of 5.6 ± 5.9 years to a mean of 4.9 ± 5.4 years over the past decade, suggesting that the medical profession may have become more aware of the features of the disorder and better at diagnosing it earlier. However, our data also showed that patients with CNO/SCCH do still make the round of a number of different specialists before being finally referred to a specialized center for rare bone diseases for diagnosis and treatment. The observed significant relationship between delay in diagnosis and extent of affected skeletal sites supports a likely role of delayed diagnosis in progression of untreated disease, and potential room for improving natural history, outcome of treatment, and thus prognosis by shortening delay in diagnosis, although this remains to be established by long‐term studies of large number of patients.

## Conclusions

CNO/SCCH is a rare autoinflammatory bone disease, characterized by distinctive clinical and radiological features. The clinical spectrum of the disorder is wide, and a comprehensive revision of its current classification is required to capture its diverse aspects, particularly in adult‐onset cases, which are to date often bundled under the diagnosis SAPHO syndrome, although not fulfilling the criteria for this. Underdiagnosis, misdiagnosis, and delayed diagnosis are still common, highlighting remaining difficulties in recognizing the spectrum of the disease across different medical specialties. In adults, long‐standing untreated disease is associated with potentially significant morbidity caused by the irreversible alteration in the structure of affected bones by hyperostosis, soft tissue ossification, and secondary degenerative changes in joints adjacent to affected bones, resulting in sometimes debilitating chronic symptoms of pain and variable impairment of shoulder girdle and spine function. These symptoms may be associated in the long‐term with a significant burden of illness and a negative impact on quality of life. In CNO, early diagnosis and timely initiation of treatment hold, therefore, significant clinical implications because they may have a significant impact on improved prognosis by potentially preventing or arresting disease progression, although this remains to be established by long‐term follow‐up studies. We hope findings from our study will represent an important building block for revision of the current nomenclature and classification of chronic nonbacterial osteomyelitis in adult‐onset CNO and will provide a tool for increasing awareness of all stakeholders involved in the care of patients with CNO/SCCH for the distinctive clinical features of this intriguing and complex rare autoinflammatory bone disorder.

## AUTHOR CONTRIBUTIONS

**Ashna Ramautar:** Data curation; formal analysis; investigation; methodology; project administration; writing‐original draft; writing‐review & editing. **Natasha Appelman‐Dijkstra:** Conceptualization; data curation; investigation; supervision; writing‐review & editing. **Shannon Lakerveld:** Data curation; formal analysis. **Marielle Schroijen:** Investigation; writing‐review & editing. **Marieke Snel:** Investigation; writing‐review & editing. **Elizabeth Winter:** Conceptualization; data curation; investigation; writing‐review & editing. **Neveen Hamdy:** Conceptualization; data curation; formal analysis; investigation; methodology; project administration; supervision; writing‐original draft; writing‐review & editing.

## Conflict of Interest

All authors state that they have no conflict of interest.

Author Contributions

*Study design*: AIER, NMA‐D, and NATH. *Study conduct*: AIER, NMA‐D, SL, MAS, MS, EMW, and NATH. *Data interpretation*: AIER, NMA‐D, EMW, and NATH. *Drafting manuscript*: AIER, NMA‐D, and NATH. *Revising manuscript content*: AIER, NMA‐D, EMW, and NATH. *Approving final version of the manuscript*: All authors. AIER and NATH take responsibility for the integrity of the data analysis.

### PEER REVIEW

The peer review history for this article is available at https://publons.com/publon/10.1002/jbm4.10490.
